# Ethical Concerns in Sport: When the Will to Win Exceed the Spirit of Sport

**DOI:** 10.3390/bs8090078

**Published:** 2018-09-03

**Authors:** Nancy Vargas-Mendoza, Tomás Fregoso-Aguilar, Eduardo Madrigal-Santillán, Ángel Morales-González, José A. Morales-González

**Affiliations:** 1 Área Académica de Nutrición, ICSa, Universidad Autónoma del Estado de Hidalgo, Pachuca de Soto CP42000, Mexico; nvargas_mendoza@hotmail.com; 2 Depto. de Fisiología, Laboratorio de Hormonas y Conducta, ENCB Campus Zacatenco, Instituto Politécnico Nacional, Ciudad de Mexico 07700, Mexico; fisiobiologo@hotmail.com; 3 Laboratorio de Medicina de Conservación, Escuela Superior de Medicina, Instituto Politécnico Nacional, Plan de San Luis y Díaz Mirón, Ciudad de Mexico CP 11340, Mexico; eomsmx@yahoo.com.mx; 4 Escuela Superior de Cómputo, Instituto Politécnico Nacional, Av. Juan de Dios Bátiz s/n esquina Miguel Othón de Mendizabal, Unidad Profesional Adolfo López Mateos, Ciudad de Mexico CP 07738, Mexico; anmorales@ipn.mx

**Keywords:** bioethics, sports, behavior, decision-making, ethical practices

## Abstract

**Background**: The need to advance and achieve success is deeply ingrained in human evolution. As a species, humans developed instincts that allowed them to survive and transmit their genes along generations. The will to win is an instinct that has been maintained in the species for millions of years. Sport is an activity as old as humans themselves and is subject to rules; **Objective**: The proposal of this work is to explore some of the most recurrent practices to achieve the athletes’ goals, and the origins and historical use of methods or substances to improve performance and its regulation, as well as to review the impact of new technologies on achieving better results and to make a proposal of what actions should be takenin order to prevent bad practices; **Methods**: A narrative literature review of ethical sports issues and decision-making was performed in the English language; **Results**: Practically all behavior with regards to the theme of sports is regulated by ethical codes that must be followed by sportspersons, as well as by everyone involved in the athlete’s healthcare and in the athlete’s administrative, marketing, and business aspects. Notwithstanding this, winning and reaping glory implies a reward far greater than fame and fortune, which can lead to poor ethical practices in athletes, as well as in interested parties who detract from the intrinsic value of the spirit of sports. The will to win could exceed the limits of what is permitted in fair-play, like the use of prohibited methods or substances; **Conclusions**: In this work, we review some of the bioethical aspects ofsports. Additionally, recommendations are offered for good practices and to prevent falling into poor ethical behavior.

## 1. Introduction

One of the most important aspects of physical exercise is normally the objective to improve one’s health, prevent diseases, leisure, recreation, etc. However, when it comes to sport, the meaning of physical exercise takes on an aspect of competitiveness. For a recreational sportsman, that is to say, one who actually plays a certain sport without putting in extraordinary effort and a great investment of time, or one whose professional life does not depend on the sport, finds in that activity, an opportunity for the development of his physical, emotional, and mental faculties. In contrast, when you become a high-performance athlete, the pressure to win produces a totally different effect. The win means becoming the center of attention for many, from the guild of athletes in that sport to the whole nation or even the world. Elite athletes become characters with a specific weight in society, permeating into spaces that other people may not reach, accessing the so-called fame and fortune in such a way that achieving victory not only alludes to be filled with glory, but also to be the best. Therefore, this cluster of expectations around the result can lead the athlete to cheat during the movement towards success. Through various ways, we have sought to improve the performance of athletes [1] by resorting to the use of ergogenic aids from the use of dietary supplements and medications [2] to using prohibited substances or methods [3] and using technology that even often goes one step ahead of anti-doping detection methods [4].

In the present paper, the objective is to provide a perspective of the situations that lead to conduct lacking in the ethical guidelines, not only by the athlete, but also by the multiple parties involved in the sport, such as the medical and health teams, therapists, trainers, directors and team owners, sponsors, relatives, media, etc. The scope and limitations of the study are relegated to the documents published in the most recent databases, as well as to the ethical codes on which the various sports disciplines are based. Despite this, it is known that there is a great lack of formal publications regarding this topic. With this work, in a sense, we hope to contribute to showing the reality around the decision-making process that links bioethics and sport and to be aware that all behavior must be seen under the guidelines of that which is considered appropriate and not appropriate, despite the expected result. This article also requests that value judgments are not made and, instead, that all the factors that intervene in order for an athlete or for those who are involved to be led to make certain decisions are taken into consideration. We see this as an opportunity to learn to take action with different points of view and to generate one’s own criteria and always assume the implied responsibility.

## 2. Bioethics and Sports

Human coexistence in society has been influenced and determined by implicit or explicit guidelines that indicate what is “good or bad”, deriving from what is defined by “morals”;theetymology of which derives from the Latin “*moris*” or behavior, which is a concrete system of beliefs, practices, and judgments of the first order on what is appropriate or inappropriate within a culture, society, or community, which dictates whether the acts of individuals can be grouped as good or bad. On the other hand, ethics, which is derived from the Latin “*ethos*”, provides the theoretical basis for assessing why something is good or bad [5].

With the latter, it is possible to state that ethics is a conscious activity of human beings that not only evaluates what is done, but also what is not done because of negligence or the lack of ethical criteria. One concept that arises from ethics is bioethics, which is considered as the systematic study of human behavior in the ambit of the life sciences and health sciences, analyzed in the light of values and moral principles. This science basically includes the relationship of human biological nature and the biological world with the formulation of policies directed toward producing the social well-being of the present and future generations. Bioethics consists of an interdisciplinary dialogue between ethics and life, whose interdisciplinary character takes as a given that this science finds support in various disciplines, from the medical to the humanist, economic, philosophical, political, and legal disciplines [5].

In turn, there arises, within the framework of bioethics, the concept of medical ethics, described since the beginning of the 19th century by the English Physician Thomas Percival. Percival combines philosophy and science; thus, he refers to a science of reason and, as in all sciences, it has as its purpose the search for truth, which is objective, unique, and independent of the observer (in this case, the medical investigator). When medical ethics is centered on the physician, how they act is studied and scored as good or poor, according to whether it is voluntary and conscious. The bases of medical ethics are focused on the following four main principles: autonomy, beneficence, no maleficence (do no harm), and justice, as well as the diverse values that comprise the guidelines for their application [5].

The term coined as sports sciences alludes to the formal scientific study of sports from various angles. It is understood as the disciplinary set oriented toward the study and understanding of sports and physical activity. Sport in itself includes the practice of an activity and is subject to determined regulations and it nearly always entertains competitive ends. Sports is, then, a human practice as old as humans themselves and that, with the passage of time, has polished the conceptualization of and the rules that determine it. The practice of a sport normally has a recreational purpose, a professional purpose, or is a means of improving health. On addressing the rules, it is understood that sport thus obeys precepts of what should and should not be done or, as mentioned previously, of what is good or bad [6].

The formal study of sports has led to the increase in the number of disciplines devoted to the investigation and application of scientific knowledge among which the health sciences can be listed. Other disciplines include Biomedicine, Pharmacology, Genetics, Nutrition, Kinesiology, Toxicology, Biomechanics, Psychology, and Physiotherapy, to mention a few. The object of sports science is for each one to achieve, from scientific bases, the increasing optimization of the multi-causal performance of the athlete. It is thus that bioethics applied to sport analyzes the decisions that, in the health sciences, are carried out by all of those involved in the health care of the patient/sportsperson, including the physician and health professionals, relatives, trainers/coaches, technical directors, team owners, referees, and legislators, to mention a few of the most important of these individuals [7].

In the global sense, bioethics reflects on the problems deriving from the scientific and technological expansion in the practice of the medical and biological sciences and their social repercussions, both present and future, which calls for the responsible management of human life.Therefore, the multi- and trans-disciplinary study of sport becomes a facilitator of the bioethical integration of scientific and professional activities, as well as in the legislative structures and decision-making with the consequent inclusion of respect, the promotion of human life and of the person, and of their human rights in the biomedical sector [8].

## 3. Ethical and Poorly Ethical Practices in Sports

Practically all behavior regarding the theme of sports is regulated by ethical codes that should be followed by the sportspersons, as well as by all of those involved in healthcare and in the administrative, marketing, and business parts. Next, we will approach some of the situations that frequently reflect evidence of what “should be” and “should not be” in the practice of sports, highlighting the participation of the physician and the health team, trainers and coaches, as well as the sportspersons themselves [6].

A variety of ethical problems in the area of the sportsperson results in the intervention of multiple interested parties in the sports franchises and, above all, in terms of the sports teams. Among these interested parties, we find the healthcare team (physicians, trainers, physical therapists, nutritionists) [9], the athletes themselves, and the board of directors (made up of technical directors or trainers, the general manager and, in the case of professional teams, the team owner or owners). In a similar fashion, relatives, sales agents, or fans may be involved as well [7].

In this respect, health is a fundamental aspect in the well-being and performance of the athlete; thus, the healthcare team is frequently under pressure in terms of the management of an injured athlete who needs to be returned to their sports activity as soon as possible. This pressure is exercised above all by the organization and, in many cases, by the sportspersons themselves. Therefore, this has led to practices that place the athlete’s health and physical integrity at risk. To mention an example, the excessive use of analgesics has been reported,whichallows the injured athlete to continue playing in a game that, at that moment, would appear to benefit the team but, in reality, would worsen the injury and in the long-term, could have negative effects, probably generating a greater cost in all respects [7,10].

Various physicians who are responsible for professional sports teams have mentioned experiencing pressure from the technical directors and team owners to make it possible for the player to return to training sessions and games with a minimal recuperation time. It is noteworthy that the sportsperson, for the most part, wants to return rapidly to their sports activity independently of the degree of injury or whether some type of surgical intervention is required. The pressure exerted by multiple parties has brought to light the use of little-recommended practices to produce a rapid recovery that can even go against the ethical practices of medical management [11].

It is a fact that the board of directors tends to exert pressure on the physician who engages in these practices and, on the other hand, on the athletes themselves to accelerate their recovery, implementing certain fear with regard to the annulment of their contracts or salaries due to the loss of time during recuperation. In this respect, it is the players themselves who pressure the physician to make use of more intensive recovery methods or techniques that, in the short-term, appear to be effective, notwithstanding that, in the long-term, these can be risky [12].

A frequently observed situation is that the players undergo not only physical, but also psychological, injuries. The response on confronting a serious injury can be very diverse in athletes. It has been heard said that for those who can take the injury as a natural situation to which any athlete is exposed to on a day-to-day basis with the practice of sports, the only thing needed is to rest and recover in order to get back into physical shape again. The athlete who perceives the injury as a disaster can undergo a strong impact psychologically. It is common for the athletes in the latter category to tend to accelerate their recovery process in order to return to their sports as soon as possible, to the degree that they find themselves forced to return to training or playing under intense pain, which makes them more respected by their team members compared to than those who decide to take more time to recuperate [1]. Those who choose to rest become prey to jokes and abuse by the other players. Therefore, gaining the respect and admiration of their teammates tends to be an incentive for the player’s ego that makes the physical suffering worthwhile, even though they have not yet recovered from their injury [13].

It is so important for a player to keep active within the team that, according to some sports medicine reports, various players have not revealed the extent of their injuries for fear that the physician would inform the trainer or technical director of the nature of their injury and that the player would be removed from the training sessions or the game [14]. The physician’s preparation and knowledge confer upon them the capacity to be aware of the situation and it is in these cases that the athlete asks the physician not to inform the trainer or technical director about the true situation. Sometimes the athlete even proposes the use of little-recommended substances or techniques to the physician in order to hasten the athlete’s recovery [3].

Team physicians also undergo pressure from the board of directors on complying with the demands in terms of management, which is, in the end, a business. Therefore, keeping the board of directors satisfied permits these physicians to possess a privileged position that affords them, up to a certain point, benefits such as status within a team of healthcare professionals. This implies the possession of special power in terms of decision-making if some type of reward or personal interest is involved outside of the context, which increases the risk of falling into marginally ethical behaviors [9,15].

The doctor/patient relationship can be influenced by the hierarchical position in the organization that physicians occupy and the relationship that they entertain in terms of the board of directors. This conflict of interest occurs at a core point concerned with the surveillance of professional ethical behavior. From this, we can derive the following questions: How should the position of the Sports Physician be structured? Are the medical ethical guidelines established for the real management of the patient/athlete updated? Ideally, the behaviors should be established through contractual obligations that take the ethical behaviors for granted, but it is uncertain how the contract exerts an influence on the physician’s duties regarding the confidentiality and well-being of the patient/athlete [10,15,16].

### 3.1. The Use of Illegal Substances and Doping

The achievement of fame, victory, and recognition was the objective of athletes in the early Olympic Games. Today, these objectives continue to comprise the most important reasons for winning for the competitors. The difference lies in that there is a reigning factor in the motivation: the money. For an elite athlete, to win a medal or international championship is a guarantee ofgenerating lucrative contracts and juicy winnings in the future, ensuring their fame at the highest level [17]. The pressure to reach or maintain this position gives rise to the recurring temptation to use prohibited methods or substances [18,19].

It is a reality that, at present, sportspersons are able to count on an immense variety of tools to improve their performance in comparison to the past. This type of support is known as “ergogenic aids”, which are defined as any training technique, mechanical artifact, nutritional practice, pharmacological method, or psychological technique that improves the performance, sports capacity, and/or theadaptations to training. It can include aids that allow the individual to prepare prior to the exercise or to improve the effectiveness of the exercise and/or in the recuperation process. These types of aids also permit the individual to tolerate more intense training, helping them to recover more rapidly, or helping them to be injury-free for as long as possible [20]. In this argument, one must distinguish the point at which it is possible to utilize ergogenic aids without crossing the line of what is not permitted to be used for the optimization of sports performances [21].

#### Historical Antecedents of Doping in Sports

Survival is a basic need of all species. Humans struggle constantly to advance and the desire for success is found deep in their desire to evolve. The will to win is an instinct that has been preserved in the species for millions of years. The drive toward achieving victory and avoiding defeat is profoundly rooted and is one of the most extraordinary aspects in the history of human evolution [22].

The most primitive instincts, such as competition, fear, finding food, and assuming risks, had to be developed millions of year ago to ensure the survival of our species. It has now been some four and a half million years ago that the first human ancestors lived and were required to develop their instincts to obtain caloric foods to survive and transmit their genes to the following generations. During this constant struggle, the instinct of competition was perfected to the degree that it was capable of providing a trigger when one waswinning; whether thatinvolves getting a job, being more prominent, or even winning an Olympic Medal, the responses are the same in any case. This marvelous instinct remains among us and is closely related tothe history of sports activities [23]. In the theory of the evolution of sports, it was established that the most popular sports among males require the development of skills necessary for success in physical competitions that are similar to hunting and events involving the struggles that primitive males underwent. On the other hand, winning athletes gain a privileged status that offers them better reproductive opportunities in ways similar to those that our ancestors obtained through hunting and triumphs in battle [24].

On understanding that humans had to engage in enormous physical efforts to hunt animals of extraordinarily great dimensions, to overtake exceedingly fast animals, and to escape from them, we know that they had to develop physical qualities and aptitudes such as strength, speed, potency, and resistance that, later, would be necessary to perform sports activities. Sport aroseas a playful activity and one of amusement, but also as a need to demonstrate who is the strongest and the fittest for survival, remembering that the most adapted human is the one who cantransmit their genes, ensuring the survival of their species [24]. For our ancestors, a key advantage was possessing competitive instincts [25].

It is surprising to see how, anatomically and physiologically, our body and brain came to mold themselves, working in harmony to provide us with a pleasant experience when we win. In a battle against an opponent, when one has the sensation of winning, the nervous system functions in such a way that our attention is at its finest, our reflexes are at their most rapid state, and our responses are at their most efficient state. Once victory is ensured, a physiological response that makes us experience the reward associated with the release of dopamine is triggered, which stimulates the brain, creating a profound sensation of pleasure and well-being. Next, endomorphins are released, which are opioid peptides that work as neurotransmitters, combatting fatigue, pain, and the sensation of euphoria, which minimizes the perception of pain due to its analgesic effect although the individual has suffered an injury, stopping the sensation of pain from reaching the medulla and the brain. The chemical discharge of adrenalin continues by means of the suprarenal glands and the release of testosterone from the gonads toward the bloodstream during the competition provides strength, helps us to maintain a state of alertness, and allows for a more rapid recuperation. Adrenalin increases the respiratory frequency and cardiac rate, ensuring the flow of oxygenated blood to the muscles and brain. This situation facilitates the individual to be found in their best physical state, disposed to face another competition. The process described above is identical in the sports contest [26].

Contrariwise, losing is a much more prolonged and painful experience than that which can arise from winning; in other words, failure feels terrible. When one has the sensation of beginning to lose the competition or struggle, the perception of tiredness, pain, and fatigue isemphasized. Chemically, there is a release of cortisol, which is a glucocorticoid released under situations of stress by the suprarenal glands that, together with adrenalin, promote the sensation of anxiety and fear. If the loss is catastrophic, it produces immobility in order to protect the brain, avoiding non-essential functions such as voluntary movement; this latter function is one that we share with reptiles. The activity of the vagus nerve is reduced and the blood flow in the gastrointestinal tract diminishes, generating the sensation of a vacuum in the mouth of the stomach. Muscles also undergo a loss of blood supply, with the loss of control of the extremities. In the brain, the hippocampus, which is the zone situated at the interior of the temporal lobe, is stimulated, which participates in important functions in long-term memory. The latter ensures that the loss is remembered forever. Additionally, the amygdala that, together with the hippocampus, forms part of the brain’s limbic system, is known as the center of the emotions. It is charged with transforming that memory into a deep sensation of sadness. All of that creates a penetrating memory of failure so that one will avoid committing the same mistake again. This is one way that the body ensures that the lesson has been learned [26].

Because we have evolved in a body that affords us a greater reward every time we compete, each time the demand is greater. The biological motivation that leads us to go further in each competition has been the instinctive development of the fear of failure. Because winning makes us feel good and losing makes us feel terrible, the instinctive fear of failure will allow us to do nearly anything to not lose [27].

In this physiological explanation, another instinct is found to be implicit: the fear of assuming risks. The risk is related toreward and searching for the best reward is part of our evaluative heritage. The greater the need, the greater the risk that one is willing to take for money, food, or anything else. Humans are derived from beings who risked, won, and continued to populate the world [27,28].

In the practice of a sport, in contrast with solely physical exercise, the physical activity developed is subject to rules and statutes with competitive ends. Therefore, it can be stated that the basis of sports was founded on the competitive instincts and those of the risks that we inherited. Socially, we have changed a great deal; biologically, we preserved our essence. Sport is also a way that we relate toour peers; it is a way of comparing our position with that of “the other”. Initially, the reward for winning was reinforced by the admiration and respect of those who witnessed the competition, while today, and as mentioned previously, money is the preponderant factor [18,19]. Thus, winning in whichever way possible is the sole objective of every competitor.

In sport, practices to ensure victory by increasing the athletes’ performance have been historically documented. From the first Olympic Games, the consumption of hallucinogenic mushrooms as stimulants was reported. From the beginning of the past century, scientific and medical interest was aroused concerning the diet and training of Olympic athletes that led to the creation of synthetic hormones such as testosterone and the use of nervous system stimulants in Olympic athletes [29]. In Table 1, there is a brief description of some of the most relevant historical antecedents with respect to the use of ergogenic substances and the creation of international organisms for the surveillance of the use of prohibited methods and substances [30].

### 3.2. The Regulation of Doping in Sports at Present

WADA [32] was created in 1999 by the International Olympic Committee (IOC) after, in 1998, a complete network of doping was discovered in elite cycling. The IOC is an independent international agency funded by the sports movement and by the governments of the world. Its main activities include scientific investigation, education, and the development of capacities to detect doping and monitor the world anti-doping code, which is a document that establishes the policies of anti-doping in sports in all of the world’s nations. The agency entertains the creation of a world in which athletes can compete in a doping-free environment as its vision.

This international organization is charged with the impartial, objective, and transparent surveillance of sports practices carried out under the values of the spirit inherent in sports established in the code. It is charged with observing the events under the highest ethical standards, avoiding improper influences and conflicts of interest that could affect its impartial and independent judgment. It is involved in developing policies, procedures, and practices that reflect justice, equity, and integrity [33].

The world anti-doping program involves all of the following elements to ensure the harmonization of the elements in the practice of national and international programs. The principal elements are as follows [34]:

1. The code.
2. The international standards.
3. The models of best practices and guidelines.

The world code of anti-doping was adopted for the first time in 2003 and set into motion in 2004. Later, it was revised and modified in the year 2009. In 2015, a review of the code was conducted. It is the reviewed code that is, at present, the code that the World Anti-Doping Agency (WADA) employs for the surveillance of doping in world sports [34,35,36]. During the last WADA meeting on 17 November 2017, the proposal was elaborated to change some of the statutes, which were entered into use on 1 April 2018. The code comprises the universal document on which the WADA program is based, and its purpose is to advance the efforts to harmonize the elements of anti-doping, which require uniformity and, at the same time, flexibility, in order to be agreed upon and implemented. The code has been designed by taking into consideration the principles of proportionality and of human rights [30,35].

The purpose of the international standards is the modulation of the world organizations charged with the operational and technical parties of the anti-doping programs. The international standards must be complied with as a mandate of the code; these standards are submitted to constant review by the consultants, association representatives, and international committees, together with other authorities. The revisions of the standards are published on WADA’s Official Page, together with the date on which they were entered into effect [32].

Models of best practices and guidelines are developed based on the code and international standards with the aim of providing solutions in the different areas of anti-doping. These documents are furnished by WADA to signatories and other interested parties, but are not obligatory: they function as recommended guidelines and can be utilized as models for organizations to design their own codes based on the WADA code. All of this information is available on the official WADA website [34,37].

Now, what is the objective of regulating the ethical principles based on the WADA program? The answer is that they seek to preserve the intrinsic value of sports, frequently called the “spirit of sports”, the Olympic spirit, the essence of Olympus, the pursuit of human excellence through perfecting a person’s natural talents. The spirit of sports is the celebration of the spirit, body, and mind and is reflected in the values that we find through sports, such as [34]:

- Ethics of the fair and honest game.
- Health.
- Excellence in performance.
- Character and education.
- Fun and good cheering.
- Teamwork.
- Dedication and commitment.
- Respect for the rules and laws.
- Self-respect and respect for the other participants.
- Courage.
- Community and solidarity.

Doping is fundamentally against the spirit of sports. Fighting against doping requires each anti-doping organization to develop and implement educative strategies and prevention programs for athletes.

WADA, in Article 1 of its code, considers doping to be positive when it incurs one of the violations established in Article 2.1 up to 2.10 [34].

The following are considered violations of the rules of anti-doping:

1. Presence of prohibited substances or their metabolites or markers in samples from the athlete.
2. Use or the intent to use some prohibited substance or method.
3. Evading, refusing, or failing in the taking or delivery of the sample.
4. Any combination of three failed tests.
5. Manipulation or the intent to manipulate any part of the anti-doping process.
6. Possession of prohibited substances or prohibited methods.
7. Trafficking or the intent to traffic any prohibited substance or prohibited method.
8. Administration or intent to administer any prohibited substance or prohibited method inside and/or outside of the competition.
9. Complicity to promote, aid, guide, encourage, conspire, cover, or any type of other complicity related to the violation of the anti-doping code.
10. Prohibited association between the athlete or some other person and the association of the responsible authorities of the code.

For their part, the prohibited substances or prohibited methods are made known each year through a list of prohibited substances on their official page. The list is updated at their meeting officiated in the month of October by the authorities and individuals active in each association involved in different countries and is valid from January 1 of each year [38].

The valid list for 2018 [37] takes prohibited substances into consideration at any time inside and outside of the competition:

**S0. Unapproved substances**. Any pharmacological substance that is not under the direction of any of the subsequent sections of the list and that is not currently approved by some governmental regulatory health authority for human therapeutic use (for example, drugs under pre-clinical, clinical, or discontinued development, designer drugs, substances approved only for veterinary use) is prohibited at any time. This considers the following substances:

- S1. Anabolic agents
- S2. Peptidic hormones, growth factors, and substances related and mimicking these
- S3. Beta agonists
- S4. Hormones and metabolic modulators
- S5. Diuretics and masking agents

This list consists of the Prohibited Methods:

- M1. Manipulation of the blood or its components
- M2. Physical or chemical manipulation
- M3. Genetic doping

During competition periods, the use of the following is strictly prohibited:

- S6. Stimulants
- S7. Narcotics
- S8. Cannabinoids
- S9. Glucocorticoids

Within the list, a heading that specifies the prohibition of the use of beta-blocking substances for sports is included. For further information about specific substances or methods, consult the extended 2018 list [37] emitted by WADA on their official website [32].

Within this frame of reference, the practice of promoting the use of substances that promote the performance of athletes without affecting health is important. One must constantly review the regulations models, as it is evident that ethical practices are not always ensured in the search for the win. Is using prohibited substances or methods to win worth the risk involved? How much greater is the need to win for those who have less? Their lives would be changed for the better if they win, of course, but the risk is greater, and the consequences can be catastrophic in all respects [39].

For elite sportspersons, the recent availability of prohibited substances that are identical to those produced by the human body seriously limits the viability of anti-doping tests [40]. Therefore, the need has arisen to design what is known as the Athlete Biological Passport (ABP), sustained in the monitoring of doping markers personalized for each sportsperson. This new paradigm supplies sufficient proof that the athlete can participate in a competition in an adequate physiological state free from substances or methods that are not permitted. At the same time, it can be useful as a platform for the regulation of the sport [41,42].

However, the use of technology has been supporting the development of innovative methods that seem almost imperceptible by the current means of detection. Such is the case of genetic doping (gene doping) that, in recent years, has emerged due to the contributions that the Genomic sciences have made to the field of medicine. Genetic doping is an elegant way of cheating in sports because this technique involves the most profound form of manipulation: genetic alteration [18]. The study of the genome has allowed human beings to identify key genes in the development of the athlete because they have a preponderant role in sports physiology. Among these genes are the erythropoietin gene (EPO gene) for the increase of the erythrocyte mass, the endorphin/enkephalin genes (endorphin/enkephalin genes) that improve pain tolerance, the vascular endothelial growth factor gene (vascular endothelial growth factor VEGF gene) that promotes angiogenesis, the growth factor similar to insulin type 1 (insulin-like growth factor type 1, IGF-1 gene) that allows the increase of the muscle mass, and the transforming growth factor (transforming growth factor-B, TGF-B gene) which inhibits myostatin, among others. The ethical dilemma arises from asking the question: to what extent is it valid to resort to these tools to achieve the desired objective? Under what criteria does the athlete, coach, or interested party determine the appropriateness of these methods? Is it worthwhile to circumvent the codes of ethics in order to win? It is a sensitive issue that occurs more frequently than you think. It is convenient to make use of the conscience and reason in making decisions that can permanently affect the future of one’s sports career if the truth comes to light [43].

## 4. New Technologies in the Sports Sciences and Their Ethical Use

The field of biomedical sciences in sports is very promising in terms of carrying out investigations and testing innovative procedures, into which large companies invest large sums of money [44]. In order to perform their tests and obtain investigative data, these corporations are able to achieve links with important sports teams, thus having an apparently reliable scientific backup because the majority of these novel technologies lack scientific information to support them. In the case of accepting the implementation of such experimentations, the physician should conduct a review of the scientific literature published to date on the new procedure or technology under study prior to administering it to the patient. Once the professional possesses the greatest possible amount of information, it is necessary to obtain the informed consent of the patient or athlete, explaining in detail all of the aspects of the procedure, and even informing the patient or athlete of the still unknown possible consequences that could occur as part of the clinical assay [45].

The sports health team is periodically bombarded with pressure to employ new devices or cutting-edge technology. One example of this is the use of the Direct-To-Consumer (DTC) Genetic Test, which claims that it is capable of identifying sports talent in children despite there not being sufficient scientific evidence to back it up [46]. In recent years, different laboratories have come to the fore by offering these tests directed toward trainers, athletes, and physicians, and parents or guardians in the case of minors. These DTC tests are used for determining gene expression when confronted with different stimuli, such as different components of the diet, which has been dominated by Nutrigenomics [47]. In the sports world, these tests have been employed to motivate athletes to consume dietetic supplements of vitamins, minerals, and other compounds that affirm that they have a significant impact on high performance. These tests consist of obtaining a sample of the oral mucosa or saliva, which can be taken easily at home and sent to the laboratory for processing. Frequently, the results obtained by the laboratories are taken advantage of to offer additional services such as personalized training programs, supplements, or supposedly individualized nutritional strategies, evidently adding additional costs to the initial payment [48,49].

Among the services that these companies have a tendency to offer, we find the possibility of discovering genes associated with increasing the sports performance and knowing the innate strengths with which the athlete is equipped, as well as their natural limitations, among others (Table 2). However, to date, the scientific evidence that supports this is extremely weak [50].

The early identification of sports talent represents a great opportunity to invest time, money, and effort focused on the innate qualities and aptitudes of the athlete, specifically in children [51]. It is known that professional sport is a highly lucrative ambition; thus, the early detection of sports talents presupposes success, in that, based on the previous genetic diagnoses, trainers or parents can plan training regiments with greater certainty. On their part, the physician and/or physical therapist can establish training regiments or therapies to optimize the rehabilitation time. The nutritionist would have the possibility of designing dietetic, hydration, and sports supplementation plans based on the genetic response of the athlete to exposure to certain nutrients or biomolecules that appear to influence sports performance, as well as the necessity to mention this to each of the professionals in their area [47].

It is true that genetic tests, after their implementation in the detection of the human genome, have come to be employed frequently in clinical use to detect certain genetic diseases and in the identification of polymorphisms that permit identifying possible risks in the development of some disease or conditions, especially those that are pathological. While costs have changed over the past two decades, the initial cost of DNA sequencing rose to various thousands of millions of dollars; currently, the cost of a DTC test is one thousand dollars. This refers to the greater accessibility of this type of technology. This accessibility opened the door to multiple areas of science to make use of biotechnologies in the generation of new knowledge and its application in practice. Of course, the sports sciences also saw their area of opportunity in the early detection of athletic qualities in children and young people with the purpose of channeling them into a sport akin to their supposedly innate sports skills, qualities, and capacities [52].

Despite the accessibility that is available today to defray the cost of genetic sequencing tests, the interpretation of these tests continues to be found in a marginally developed phase. International ethics committees suggest that the tests be submitted to evaluation in terms of different areas, such as their analytical validity, their validity, and their clinical usefulness such as the social, ethical, and legal implications that their use involves [53].

At present, there is, to our knowledge, no international regulation that is charged with reviewing the validity and the use of the tests legislation, which varies from country to country. While in countries such as France, Switzerland, Germany, and Portugal, it is established that genetic tests can only be carried out by a specialized medical team; in the U.K., there is no such regulation yet. In the European Union (EU), a regulatory proposal was recently evaluated for DTC tests, in which it was established that companies should supply scientific evidence of the clinical validity of their tests and that these will need specialized medical supervision. On the other hand, in the U.S., a report from the Senate of the Government Accountability Office (GAO) mentions that a genetic test for the Food and Drug Administration (FDA) is considered a medical test if it is manufactured as a kit and sold by a laboratory. In such a case, it is the laboratory that decides whether the tests possess sufficient validity according to the purpose with which it is desired to be utilized. Despite all of the laboratories following the Clinical Laboratory Improvement Amendments of 1988 (CLIA) and complying with all of the quality standards, they still do not have, to our knowledge, a specific regulation for the genetic tests. Therefore, the lack of regulation under this heading remains a problem. It is quite interesting to come to know that there have been mentions of the same DNA samples belonging to the same individual but with distinct names being analyzed and, on being sent to the laboratory, were determined to have different genetic variants. With respect to physical exercise or sports, the results of these tests lend themselves to the recommendation that the use of supplements is overrated, that supplements have not even been tested scientifically, and that these may even be harmful to one’s health [46].

Thus, the results of these tests can generate implications at the psychological, social, and economic level beyond that of health. First, the expectation generated by obtaining a result in favor of certain innate skills and qualities could be harmful if, after receiving the test, the expected performance is not observed in the subject after being informed of this information and structuring the subject’s training sessions to be channeled toward what was indicated on the test. This would imply psychological consequences in the child, as well as changes in self-esteem and social stigma. In the case of children who are not developed for some sports discipline, there can be frustration on the part of their parents or guardians. Above all, ifthe child’s parents were professional sportspersons, they may decide to have the test conducted to ascertain with greater certainty whether to enroll their child in a specific sport. However, in the end, the child will probably not present the desired results in their development as an athlete because perhaps, simply, their interest lies in other activities and not precisely in that specific sport. Therefore, the parents should be aware that, although they are responsible for decisions concerning their children, they also need to consider the tastes, interests, and opinions of their children. Contrariwise, they will oblige their children to engage in sports activities that imply physical effort, personal sacrifices, and the investment of time that, instead of benefiting them, could cause irreparable damage to their integrity and that could even be considered as a violation of their rights as children [14].

Current scientific evidence coincides in the study of the genes ACTN3 and ACEI [54], with which a relationship has been reported with sports development, and it is precisely these genes that are most analyzed and that are offered by the laboratories in their genetic tests [55]. In spite of this, meta-analyses up to 2017 indicate that there is an association between the genetic variant ACE II and physical performance, especially in resistance, while the genetic variant ACTN3 RR is associated with performance in the sports aspects of speed and power [56]. Despite this information, on reviewing the strength of the association, the results were found to be insufficiently significant [57]. A large part of the existing studies has been carried out in other regions of the world. Solely in U.K. databases, it has been reported that there are around 20 million persons, representing one-third of the total population, with an ACTN3 RR genetic variant, and only a very small portion of these have become high-performance athletes. This means that it is not sufficient to have the genetics, but a favorable environment is also required for sports development [58]. In a study conducted on sprinters of Chinese origin, it was found that the polymorphism ACNT3 R577X exerts an influence on the power and speed of the elite athletes. In a meta-analysis that was carried out in Japanese athletes [59], an association was confirmed between the RR + RX genotype of the polymorphism ACTN3 R577X and athletes who practice speed and power. In addition, a relationship was confirmed between the ACTN3 RR + RX genotype and the performance of long-distance athletes [49]. In Italian athletes, the association of the ACTN3 R577X polymorphism and the performance level has not been proven [48]. As can be observed, the results of the studies conducted among athletes are not conclusive in terms of the association of genetic variants and sports performance.

Another aspect to consider is the enormous genetic variability that exists among ethnicities. Therefore, it would not be appropriate to consider the results of the tests as an absolute if there wereno genetic databases in the country-of-origin for comparison. In any case, it would be more accurate to know the genetic variants related to sports performance by ethnicity; thus, we would perhaps have a less uncertain diagnosis [47].

Bearing the latter in mind, it must be said that companies that market DTC genetic tests cannot guarantee absolute results based on a sole genetic variant (the most studied variant), which cannot be absolutely proven in all athletes in terms of training and, in any case, regarding the sports destiny of an individual [47]. Despite this, various companies report the study of up to 27 genetic variants linked with physical performance. In practice, however, the level of scientific evidence that backs any described polymorphism has been very weak or nonexistent to date [46].

What are the ethical and legal guidelines to consider when carrying out one of these tests and, above all, when making important decisions on the future of a person in sports? At present, there is a consensus in the medical scientific community that states that genetic tests should only be carried out after the interested party has given their informed consent, which can only be proven when the patient has received all of the necessary and relevant information about the process and the risks, benefits, limitations, and the implications of the test. The patient or consumer should have the security that their data will not be utilized forpurposes different from those that have been informed, that the test will be performed by a trained person, and that the interpretations of the results will be analyzed by specialized genetic physicians [46,60].

In relation to testing children, the American Society for Human Genetics recently published that DTC genetic tests in children are not recommendable until the companies offer the service of ensuring the quality, specificity, and validity oftheir tests and until there is adequate consulting before and after the process. Genetic information is potentially sensitive and requires the assurance of the highest level of safety and confidentiality [61]. Any relevant personal information of the subject under study should be protected and should not be shared with other intents without the consent of the person implicated, according to the current data protection laws. It is necessary to promote reforms in the law with respect to punishing companies that do not strictly comply with the latter [46].

## 5. Confidentiality between the Health Professional and the Athlete

Among the ethical codes of health professionals, there is an established obligation of confidentiality that the professional owes to their patient, except in conditions under which safeguarding the information implies some risk for the patient or for third persons. However, the frequently occurring question is, to what point is it permissible for the board of directors or the sports committee to know the medical situation of an athlete without violating this aspect of confidentiality? [10].

Certainly, a trainer or even the board of directors has a legitimate right to know the medical situation of a new sports talent. Having a record of the past or present relevant medical history is important in order to know the possible susceptibility of the athlete and, in this manner, prevent injury or harm to the athlete. However, in contrast, there is something known as confidential information, concerning the personal history of the athlete. When this information does not interfere with the athlete’s sports performance, the physician is not obliged to inform the trainer or directors of this information. Nonetheless, the physician is not forced to maintain confidentiality when the athlete engages in inappropriate behavior, for example, upontheir consuming some type of prohibited substance to improve their performance [62]. In this manner, the athlete must be aware that, due to the nature of the process of strict confidentiality, the latter is not guaranteed and even at the beginning of each evaluation, the physician should inform the athlete of this.

With respect to the theme of confidentiality, some reports speak of the management of highly sensitive information on the part of the physician in which the athlete had requested strict confidentiality under his right within the doctor/patient relationship. Despite this, the physician had had to report to the trainer or the board of directors on this event for the good of the athlete, as well as for the good of the interested parties. Next, we list some of the most frequently observed situations [11]:

1. Some member of the team has some type of infection such as hepatitis or HIV.
2. Some member of the team has some type of disease that requires specific medication and that renders the practice of the sport somewhat dangerous.
3. Some member of the team is taking some type of drug for recreational purposes.
4. Some sportsperson is pregnant and does not wish the board of directors to know her situation for fear of losing her place on the team.
5. Some member of the team is taking some medication to relieve pain despite the indications of the physician based on the knowledge that the drug can cause greater harm.
6. Some member of the team is planning to take or has voluntarily been taking some prohibited substance to improve their performance without the trainer or physician knowing about this.

Another type of pressure with regard to confidentiality is that exercised by the communications media, in the case of professional athletes, who approach the physicians or health teams in charge to obtain information on the status of the athlete/patient. There have been cases in which some physicians who have fallen into behaviors lacking in ethics have provided confidential information on sportspersons in exchange for economic remuneration or publicity for their services. Likewise, privacy is a very sensitive topic that cannot always be ensured due to the accessibility of the media of the sports installations, whether private or public, whichthe athlete attends [10].

The athletes must be duly informed previously of investigations being carried out that obtain information on the athlete. The individual must give their approval of the investigation and obtained information through informed consent, after which the purpose and use of the patient’s personal medical information should be explained in detail. In conducting such an investigation, there should not be an incompatibility of interests between the investigators and the sources of financing. The evaluating ethical committees responsible for the supervision and approval of the protocol should remain attentive should this arise and, were this the case, it would not be appropriate to confer their consent for the development of the protocol. Studies should be performed and reported on under the aegis of strict honesty and impartiality [7,10].

## 6. Recommendations for Maintaining Ethical Conduct in Sports

First, on many occasions, there is a tendency toward thinking that, in fact, the health team, after having received a professional formation, knows the ethical principles with which they are to direct their practice because it is known that the ethical codes are reviewed as part of the curriculum of each profession. In any case, it is necessary to offer instruction in the basic principles and concepts of ethics, as well as to constantly receive feedback on the ethical codes deriving from the models of appropriate behavior in professional conduct. Notwithstanding this, one must bear in mind that ethical codes are only frameworks to be used as a reference for decision-making and that they do not offer a specific guideline for the resolution of all ethical problems and dilemmas [13,16,63].

Ethical principles in the medical practice determine that the health professional should act in a manner that always seeks the patient’s good interest (athletic or not), even if there is some reward or a position of incentive involved. Thus, there should be key rules for the relationships among physicians, athletes or teams, and organizations, to avoid the physician of the health team from entertaining some interest different from the health and well-being of the athlete in favor of receiving some title, benefit, or the acknowledgment of some favor from the organization or board of directors. In this fashion, the diagnoses and the medical decisions should come under the surveillance of the corresponding ethical committees and medical associations [63,64].

It is important to utilize different reasoning criteria as part of the patient’s care. Prior to making any decision, it is pertinent to listen to the opinion of other professionals who are not involved in the particular case, with the objective of knowing other viewpoints and suggestions on what should be done. In this way, the decision-making on the part of the health team should be founded on professional judgment, always seeking a balance among the ethical principles of the patient’s right to autonomy, the obligation of the professionals to avoid doing harm, generating well-being, conducting a risk-benefit assessment and, at the same time, attempting to satisfy the needs of the interested parties, such as the trainers, the technical directors, and the board of directors [63].

The health team should share the cases and experiences undergone by their fellow health professionals through the diffusion of information in the form of scientific publications and in spaces such as congresses, symposia, or assemblies of experts in such a way that the discussion invites self-reflection in order to help them to solve problems and to establish solutions for future ethical dilemmas [10,65].

The success of the practice of sports medicine and sciences does not only depend on the skills to render a diagnosis and select the appropriate medical treatment, but also onthe making of ethical decisions. Therefore, the health team should be trained in confronting ethical problems in their professional practice [65,66].

## 7. Conclusions

In human nature, there will always exist the emotion of getting there first, of avoiding the agony of losing, and the reigning desire for victory; all these are the product of a unique selection of competitive instincts. We have also developed the ability to group ourselves together with the largest brain, which has afforded us the possibility of being a highly competitive species. In the practice of sports, it is clear that to achieve success, it is necessary to make use of these instincts; however, the road to success is paved with ethical principles that signify the appropriate behavior of the sportsperson, as well as that of all other interested parties. To disregard these principles on which ethical codes are sustained is to fail to recognize and to go against the spirit of sports.

## Figures and Tables

**Table 1:** The historic events concerning the use of performance-enhancing substances.

Date	Event
**776. A.C.**	The use of hallucinogenic mushrooms is reported in athletes performing in the first Olympic Games.
**1922**	Scientific and medical interest begin to arise concerning the diet and training of Olympic athletes.
**1930**	The first synthetic hormones are developed.
**1935**	The hormone testosterone is synthesized for the first time.
**1936**	The use of anabolic steroid hormones and amphetamines on athletes in the Olympic Games in Berlin, Germany is reported.
**1952**	An athlete dies during a competition in the Olympic Games due to the use of stimulating substances.
**1960**	In the Olympic Games in Rome, Italy, the International Olympic Committee (IOC) prohibits the use of anabolic steroids.
**1961**	The IOC establishes a medical commission to review the use of prohibited substances.
**1968**	The first tests are implemented for the control of doping for detecting analgesics, opioids, and amphetamines in the Olympic Games in Mexico City.
**1971**	The provisions for the doping tests were prescribed as a mandate in the IOC Letter.
**1974**	The use of anabolic steroids is prohibited by use of the gas chromatography test. In the same year, the IOC includes these in the list of prohibited substances.
**1988**	The “Festina Case” in the Tour de France against doping in elite cycling discovered an extensive network of international doping of undetectable substances as follows: Testosterone, erythropoietin, and the growth hormone.
**1999**	The World Anti-Doping Agency (WADA) was established for the professional management and control of doping.

Modified from Hwang, et al. 2014 [30,31].

**Table 2:** The services offered by laboratories and the athletic skills described by the Direct-To-Consumer test [46,50].

Services Offered by Laboratories	Athletic Skills Described by DTC Tests
- To discover how genes can contribute to sports aptitudes	- Design of programs of body-fat loss and the increase of muscle mass
- To take advantage of the innate strengths and to know the biological limitations	- Manipulate the regulation of the blood supply, work capacity, and muscular metabolic processes
- Personalized training sessions based on the test result	- Increase the availability of oxygen and of energy in the cells
- Design of the training sessions in the search for sports development	- Increase the availability of muscular energy during physical exercise
- To offer parents or trainers with information on the genetic predisposition of young people for direction and to take advantage of aptitudes in different sports categories: individual sports, team sports, strength-speed sports, or resistance sports	- Prevent or delay the appearance of muscle fatigue - Design of personalized dietetic plans elaborated from the results that promise to create exercise regiments that have a better effect on the level of body composition or sports performance
- Qualitative and quantitative description of the types of muscular fibers (fast or slow) and to channel training sessions or sports aptitudes	- Improve the response from the consumption of substances deriving from dietetic supplements or drugs during the sports performances
